# Distribution of Hydrogen and Defects in the Zr/Nb Nanoscale Multilayer Coatings after Proton Irradiation

**DOI:** 10.3390/ma15093332

**Published:** 2022-05-06

**Authors:** Roman Laptev, Ekaterina Stepanova, Natalia Pushilina, Leonid Svyatkin, Dmitriy Krotkevich, Anton Lomygin, Sergei Ognev, Krzysztof Siemek, Aleksandr Doroshkevich, Vladimir Uglov

**Affiliations:** 1 Division for Experimental Physics, National Research Tomsk Polytechnic University, 634050 Tomsk, Russia; enstepanova@tpu.ru (E.S.); pushilina@tpu.ru (N.P.); svyatkin@tpu.ru (L.S.); dgk7@tpu.ru (D.K.); lomyginanton141@gmail.com (A.L.); soo1@tpu.ru (S.O.); 2 Department of Structural Research, Institute of Nuclear Physics Polish Academy of Sciences, 31342 Krakow, Poland; krzysztof.siemek@ifj.edu.pl; 3 Dzhelepov Laboratory of Nuclear Problems, Joint Institute for Nuclear Research, 141980 Dubna, Russia; 4 Frank Laboratory of Neutron Physics, Joint Institute for Nuclear Research, 141980 Dubna, Russia; doroh@jinr.ru; 5 Department of Solid State Physics, Belarusian State University, 220006 Minsk, Belarus; uglov@bsu.by

**Keywords:** nanoscale multilayer coatings, H^+^ irradiation, density functional theory, positron annihilation, radiation defects

## Abstract

Radiation damage is one of the significant factors limiting the operating time of many structural materials working under extreme conditions. One of the promising directions in the development of materials that are resistant to radiation damage and have improved physical and mechanical properties is the creation of nanoscale multilayer coatings (NMCs). The paper is devoted to the experimental comprehension of changes in the defect structure and mechanical properties of nanoscale multilayer coatings (NMCs) with alternating layers of Zr and Nb under irradiation. Series of Zr/Nb NMCs with different thicknesses of individual layers were fabricated by magnetron sputtering and subjected to H+ irradiation. The evolution of structure and phase states, as well as the defect state under proton irradiation, was studied using the methods of high-resolution transmission electron microscopy (HRTEM), X-ray diffraction analysis (XRD), glow discharge optical emission spectroscopy (GDOES), and positron annihilation spectroscopy (PAS). The layer-by-layer analysis of structural defects was carried out by Doppler broadening spectroscopy (DBS) using a variable-energy positron beam. To estimate the binding energy and the energy paths for the hydrogen diffusion in Zr/Nb NMCs, calculations from the first principles were used. When the thickness of individual layers is less than 25 nm, irradiation causes destruction of the interfaces, but there is no significant increase in the defect level, the S parameter (open volume defects amount) before and after irradiation is practically unchanged. After irradiation of NMC Zr/Nb with a thickness of layers 50 and 100 nm, the initial microstructure is retained, and the S parameter is significantly reduced. The GDOES data reveal the irregular H accumulation at the interface caused by significant differences in H diffusion barriers in the bulk of Zr and Nb multilayers as well as near the interface’s region.

## 1. Introduction

Structural materials that can withstand high irradiation doses are of great importance for modern and advanced nuclear energy systems [1,2]. Irradiation of structural materials results in the creation of a large number of vacancies and interstitial atoms, which agglomerate with the formation of dislocation loops, stacking fault tetrahedra, or nanovoids. These defect agglomerates contribute to the development of swelling, hardening, amorphization, and embrittlement, which, in turn, lead to accelerated material destruction under irradiation [3,4,5]. Currently, various approaches are being developed to improve the resistance of materials to irradiation. In particular, the formation of advanced grain boundaries, interfacial boundaries, or free surfaces (in porous structures) is one of the approaches to the rise in radiation resistance, which is currently being actively studied [6,7,8,9,10]. Most grain boundaries and interfaces are effective point defect sinks [11,12,13]. It was shown in [14] that the radiation damage of austenitic stainless steels diminishes with decreasing grain size since the grain boundaries actively capture radiation defects. Moreover, nanostructured metals, with a large specific length of boundaries, show increased resistance to irradiation compared to their coarse-grained counterparts. [14,15]. Theoretical studies have confirmed the ability of grain boundaries to capture point defects and vacancy clusters caused by cascade knockout, in both FCC and BCC metals [9,16,17]. Multilayer functionally graded coatings with different crystal structures are considered potential materials with high resistance to radiation defects since vacancy-type defects and interstitial atoms recombine at the interfaces [6,7,8,18]. Based on this concept, metals with different crystal structures (BCC, FCC, and HCP) are considered for the fabrication of multilayer nanoscale coatings (NMCs) with high radiation resistance. Studies of nanoscale multilayer coatings have shown that incoherent and semi-coherent interfaces are also a sink of point defects. Moreover, developed interfaces (as well as dislocations and vacancy-type defects) also have a significant impact on the main physical properties of metallic materials [19,20,21,22,23,24,25,26,27,28,29,30,31]. In these multilayer nanocomposites, significantly fewer defects are formed, compared with single-layer coatings under identical ion bombardment conditions [32,33,34,35,36]. However, incoherent and semi-coherent nanocomposite interfaces with different crystallographic orientations, compositions, and structures are likely to have different absorption efficiencies. The aim of the paper is to experimentally study changes in the microstructure and properties of nanoscale multilayer coatings with alternating layers of Zr and Nb after irradiation.

## 2. Materials and Methods

Samples of nanoscale multilayer coatings (NMC) with alternating layers of Zr and Nb were fabricated by magnetron sputtering on a specialized installation developed at The Weinberg Research Center, National Research Tomsk Polytechnic University (Tomsk, Russia). Single-crystal silicon substrates with the (111) orientation were fixed inside the experimental chamber with an axial rotation system. The residual pressure in the chamber was 0.002 Pa; the coatings were deposited in an Ar atmosphere at a working pressure of 0.3 Pa. Before deposition of coatings, the substrates were cleaned with Ar ions for 30 min at a voltage of 2.5 kV and an ion current of 2.5 mA [37]. Several series of Zr/Nb NMC samples with individual alternating layer thicknesses of (1) 10 nm, (2) 25 nm, (3) 50 nm, and (4) 100 nm were prepared. The total coating thickness for all samples was 1.1 ± 0.2 µm.

NMCs were irradiated with a quasi-perpendicular proton beam with an energy of 1720 keV at the EG-5 electrostatic Van de Graaff accelerator at Neutron Physics Laboratory (Joint Institute for Nuclear Research, Dubna, Russia); the radiation dose was 3.4⋅10^15^ ions/cm^2^. A~33 μm aluminum energy degrader was used to achieve the required proton distribution.

An analysis of the effect of implanted ions on the defect structure of functionally graded nanosized Zr/Nb metal multilayer systems was carried out using the SRIM-2013 software package [38]. The simulation was performed for multilayer Zr/Nb systems with different thicknesses of individual layers indicated above. The total number of falling particles was 5⋅10^5^; a proton beam with an energy of 1720 keV was directed perpendicular to the surface through an aluminum absorber 33 μm thick. According to the SRIM simulation, the indicated parameters of proton irradiation allow obtaining the Bragg peak, the maximum of which is in the region of ~85 ± 30 nm.

The layer-by-layer analysis of structural defects was carried out using the Doppler broadening spectroscopy (DBS) using a variable-energy positron beam at the JINR, DLNP in Dubna, Russia. A monoenergetic positron beam with a diameter of 5 mm and an intensity of 10^6^ s^−1^ was used. The energy range of implanted positrons was from 0.1 keV to 30 keV. The mean positron implantation depth (corresponding to these energies) was determined from the positron implantation profile for a monoenergetic positron beam in a semi-infinite solid, which is described by the Makhovian profile [39]. Annihilation γ-radiation was recorded by high-purity germanium detector GEM25P4-70 (AMETEK ORTEC, Oak Ridge, TN, USA) with an energy resolution of 1.20 keV, interpolated for the 511 keV line. The obtained DBS spectra were analyzed by determining the S and W parameters using the SP-11 software [40]. The parameter S is defined as the ratio of the area under the central part of the annihilation line to the total area of the given peak. It characterizes the annihilation of positron–electron pairs with a small momentum, which occurs mainly in void-type structural defects in the crystal lattice. A higher value of this parameter reflects an increase in free volume due to an increase in the size of vacancy type defects or their concentration. The W parameter allows one to recognize the chemical environment of the annihilation site.

The study of the structure and phase state was carried out using the methods of X-ray diffraction analysis, scanning (SEM), and transmission electron microscopy (TEM). The phase structure was investigated using an XRD-7000S diffractometer (Shimadzu, Japan) in the Bragg–Brentano geometry in the angle range of 20–75° and at a scanning rate of 5.0 deg/min. A detailed study of the sample fine structure was performed using a JEM-2100F microscope (JEOL, Akishima, Japan). Samples were prepared for TEM by ion thinning using an Ion Slicer EM-09100IS (JEOL, Akishima, Japan). Argon was used as the working gas during sample preparation; the accelerating voltage was 8 kV, the etching angle was 1.5–4, and the process was controlled using a CCD camera. Nanohardness and Young’s modulus were measured using a Table Top Nanoindentation system (CSM Instruments, Peseux, Switzerland). Measurements were performed for the load of 5 mN and exposure time of 30 s. The depth profiling was carried out on GD-Profiler 2 (Jobin Yvon Emission Horiba Group, Palaiseau, France) at a 4 mm anode with the following parameters: pressure—650 Pa; power—20 W; frequency—1 kHz; duty cycle—25%. Additionally, the electrical resistivity of unirradiated and irradiated Zr/Nb NMCs was measured [41].

**Figure 1:**
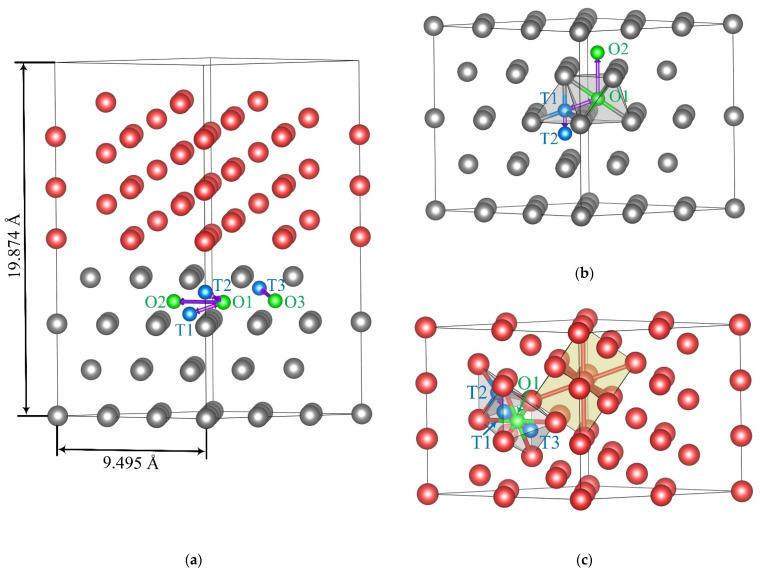
The supercell of (**a**) Zr_36_Nb_36_ multilayer structures, (**b**) α-zirconium, and (**c**) niobium. Zirconium atoms are gray, niobium atoms are red, octahedral sites are green, and tetrahedral sites are blue. The directions of hydrogen diffusion jumps are indicated by violet arrows.

The binding energy and diffusion barrier energy path for a hydrogen atom in Zr/Nb NMCs were theoretically investigated from the first principles. All calculations were performed within the framework of the density functional theory using the optimized norm-preserving Vanderbilt pseudopotential [42], realized in the ABINIT software package [43,44]. To describe the exchange and correlation effects, the generalized gradient approximation in the form of Perdew, Burke, and Ernzerhof [45] was used. The interface between metal layers was formed by Zr (002) and Nb (111) surfaces. The Zr and Nb slabs consist of four and nine atomic layers, respectively (Figure 1a). The relaxation of metal atoms was carried out in the two zirconium atomic layers and four niobium atomic layers closest to the interface. The relaxation was considered complete when the forces acting on the atoms were less than 25 meV/Å. To carry out the structural optimization and relaxation of the Zr–H and Nb–H system for discussion, a cell with 36 Zr (Figure 1b) or 36 Nb (Figure 1c) atoms and 1 H atom was adopted, and the k meshes were chosen to be 3 × 3 × 3 for the HCP and BCC structures. For Zr_36_Nb_36_ multilayer structures, k meshes were chosen 3 × 3 × 1. The cutoff energy for the plane-wave basis was set to 820 eV.

The lattice parameters calculated for pure Zr and Nb were $a_{\mathrm{Zr}}$ = 3.228 Å, $c_{\mathrm{Zr}}$ = 5.195 Å, and $a_{\mathrm{Nb}}$ = 3.292 Å, relatively, which agrees well with the experimental results [46]. To form the supercell of the Zr_36_Nb_36_ slab, the theoretical lattice parameter $a_{\mathrm{Nb}}$ of pure Nb was increased, and the lattice parameters $a_{\mathrm{Zr}}$ and $c_{\mathrm{Zr}}$ of pure Zr were reduced so that the total supercell energy became minimal. As a result, in the supercell, the Zr slab has parameters *a* = 3.165 Å and *c* = 5.160 Å, while the Nb slab has parameter *a* = 3.341 Å. The distance between Zr (002) and Nb (111) atomic layers in the interface was optimized and was equal to 2.209 Å.

According to the results of [47,48], there are four non-equivalent energetically favorable diffusion barriers for a hydrogen atom in the Zr_36_H solid solution (Figure 1b). In particular, there are two barriers along the hexagonal axis of α-Zr: between the T1 and T2 sites and between the O1 and O2 sites. There is also one barrier between the tetrahedral T1 and octahedral O1 sites (and vice versa). In the case of the Nb_36_H solid solution two non-equivalent diffusion jumps can be distinguished (Figure 1c): between the T1 and T2 sites and between the T1 and T3 sites through the O1 site. According to the result of [46], it is energetically more favorable for hydrogen atoms to occupy interstitial sites in the zirconium atomic layer nearest to the Nb/Zr interface. As a consequence, in this paper, several non-equivalent diffusion jumps were considered only in the zirconium atomic layer nearest to the Nb/Zr interface (Figure 1a). The computation of the minimum energy path between all the interstitial sites was carried out using the nudged elastic band method [49]. When the hydrogen atom was shifted along the line of diffusion jump all the metal atoms were fixed in their relaxed positions corresponding to the initial hydrogen position.

## 3. Results and Discussion

**Figure 2:**
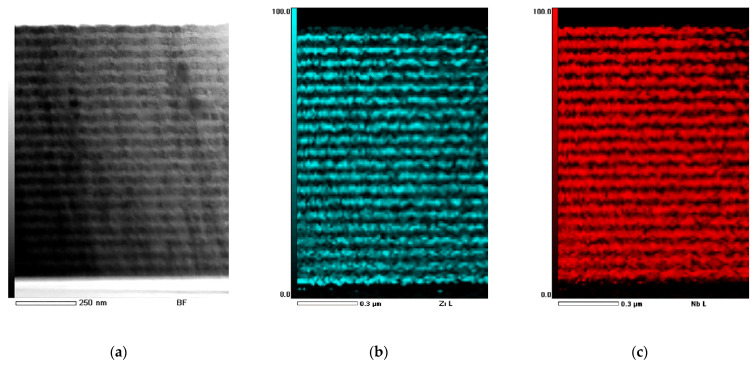
Cross-section TEM image (**a**) and EDS-mapping of Zr (blue) (**b**) and Nb (red) (**c**) layers of the as-deposited Zr/Nb NMCs.

A typical scanning TEM image and the corresponding EDS mapping of the Nb/Zr NMC cross-section in the as-deposited state are shown in Figure 2 on the example of a coating with a thickness of individual layers of 25 nm. It can be seen from Figure 2 that the deposition modes mentioned above allowed forming NMCs with alternating layers of zirconium and niobium and clearly distinguishable boundaries.

**Figure 3:**
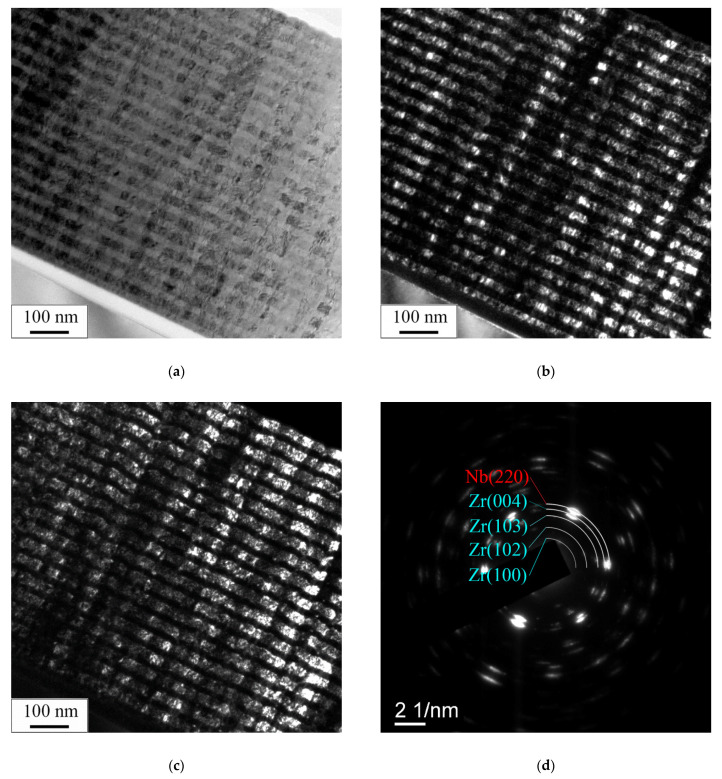
TEM images of the microstructure of the Zr/Nb NMC cross-section on the example of coatings with individual layer thickness 25 nm: bright-field image (**a**), dark-field images in (004) Zr_α_ reflection (**b**), and (220) Nb_β_ reflection (**c**), as well as corresponding SAED (**d**).

TEM studies of the transverse sections of the as-deposited coatings showed that Zr/Nb NMCs with typical structures and different thicknesses of individual layers were formed as a result of deposition. The electron-microscopy images of the Zr/Nb NMCs with a thickness of individual layers of 25 nm are demonstrated in Figure 3 as an example of the typical structure of the as-deposited NMCs. This more detailed study of the transverse sections of the as-deposited samples also confirms the formation of NMCs with alternating layers of zirconium and niobium, the thickness of which was equal to 25 ± 5 nm. In this case, nanoscale columnar grains with sizes of 10–25 nm were observed in the bulk of the Nb and Zr layers. Grains in layers grew perpendicular to the substrate. From the corresponding selected area electron diffraction (SAED) patterns (Figure 3c), the presence of an appreciable number of reflections distributed over a circle is clearly seen. The SAED patterns contained reflections from the different planes of the α phase of Zr and reflections of the (220) Nb_β_ plane. At the same time, the grain sizes for Zr/Nb 10/10, Zr/Nb 50/50, Zr/Nb 100/100 were in the range of (5–10) nm, (20–50) nm, (20–50) nm, respectively.

**Figure 4:**
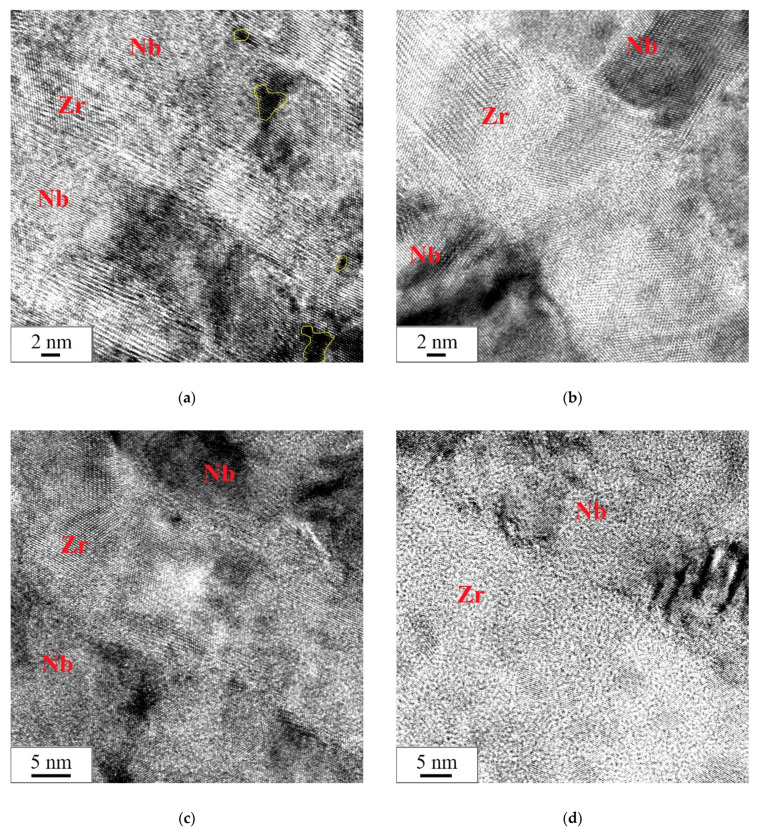
The high-resolution TEM images of the microstructure of the Zr/Nb NMC cross-section after radiation: (**a**) Zr/Nb 10/10, (**b**) Zr/Nb 25/25, (**c**) Zr/Nb 50/50, and (**d**) Zr/Nb 100/100.

The high-resolution TEM studies showed the retention of the layered structure of the Zr/Nb NMCs for all samples after irradiation (Figure 4). Irradiation at the studied doses did not affect the crystal structure and grain orientation inside the Zr and Nb layers. A feature of the samples with a thickness of individual Zr/Nb layers of 10 nm was the presence of nanovoids consisting of several vacant atomic positions (Figure 4a). Similar structural defects were observed by Callisti et al. [50]. For samples with individual layer thicknesses of 25, 50, and 100 nm, the Zr/Nb layer boundary was semicoherent (Figure 4b–d), while the layer boundaries in Zr/Nb 10/10 samples were coherent.

**Figure 5:**
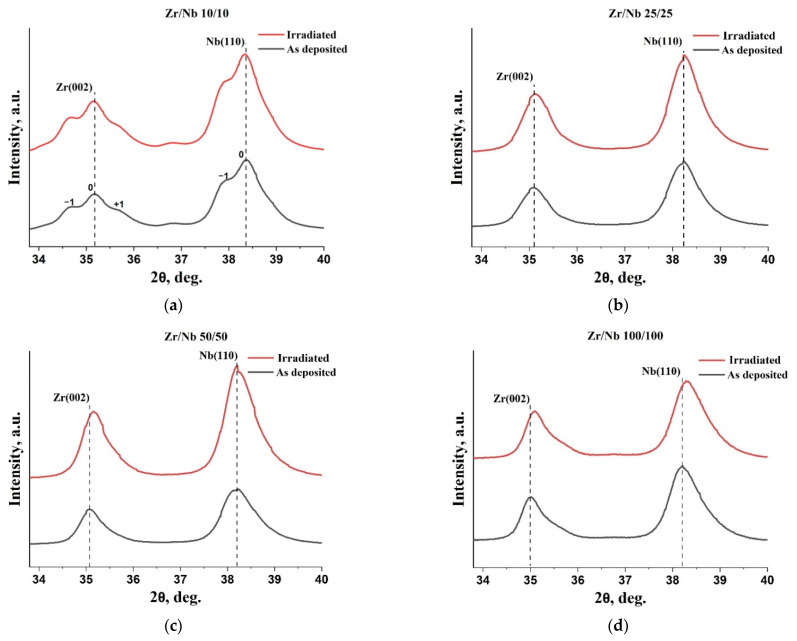
XRD patterns of as-deposited and irradiated Zr/Nb NMCs with individual layer thicknesses varying from 10 to 100 nm: (**a**) Zr/Nb 10/10, (**b**) Zr/Nb 25/25, (**c**) Zr/Nb 50/50, and (**d**) Zr/Nb 100/100.

XRD studies showed that the obtained Zr/Nb multilayer coatings had a well-defined texture. Regardless of the individual layer thickness, all multilayer coatings were characterized by (002) Zr and (110) Nb textures (Figure 5). For a multilayered system with an individual layer thickness of 10 nm, the presence of first-order satellite peaks near the main Zr (002) and Nb (110) peaks was noted (labeled as ±1 in Figure 5a), which indicates the formation of Zr and Nb sublayers with increased coherence [50,51]. Asymmetry in Zr (002) satellites and the absence of +1 Nb (110) satellite can be related to deformation and distortion of the interfaces between layers [52,53,54]. After irradiation, minor changes in the diffraction pattern were noted for Zr/Nb NMCs, with an individual layer thickness of 50 and 100 nm. For these coatings, a shift in the diffraction peaks toward higher angles was observed; for Zr/Nb 50/50, the shift occurred only for the Zr (002) peak (Figure 5c), and for Zr/Nb, 100/100 for both Zr (002) and Nb (110) peaks (Figure 4d). Diffraction peak shifting in irradiated metallic multilayers was observed in a variety of earlier studies [55,56] and is commonly attributed to lattice distortions and stresses that occurred in layers.

**Figure 6:**
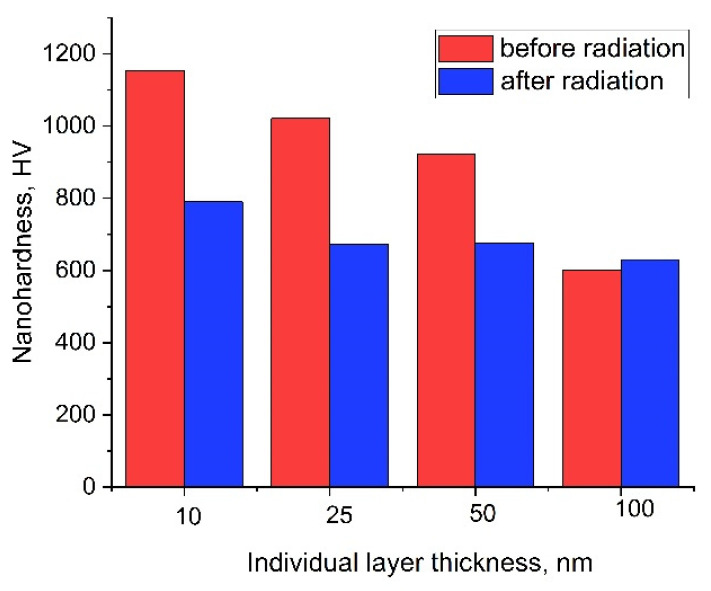
The nanohardness of samples with the different thicknesses of individual layers before and after irradiation.

It was established that samples with a thickness of individual layers of 10 nm were characterized by the highest nanohardness (Figure 6). In this case, the nanohardness of the samples was equal to 1150 ± 30 HV. A gradual increase in the thickness of alternating Zr/Nb multilayers up to 100 nm leads to a significant decrease in the values of sample nanohardness. Thus, for samples with a thickness of individual alternating layers of 100 nm, the nanohardness is 600 ± 27 HV. The main factors leading to a rise in the nanohardness of the samples with a growth in the thickness of the layers can be attributed to the Hall–Petch effect, deformations, and blocking of dislocations at the interfaces between the layers.

The Young’s modulus (E) of the studied samples statistically did not change with the thickness of the individual layers. Thus, for samples with a thickness of individual layers of 10 nm, E was (170 ± 20) GPa, for samples with a thickness of individual layers of 25 nm, it was (160 ± 10) GPa, and for samples with a thickness of individual layers of 50 nm, it was (170 ± 10) GPa. At the same time, for samples with a thickness of individual layers of 100 nm, Young’s modulus was observed to be noticeably lower and equal to 140 ± 10 GPa.

After irradiation, values of nanohardness decreased almost for all samples. Nanohardness of samples with a thickness of individual layers of 10 nm decreased by 32%, for a thickness of 25 nm, by 30%, and for a thickness of 50 nm, by 25%. For samples with a thickness of individual layers of 100 nm, the value of nanohardness after irradiation remained practically unchanged. Meanwhile, irradiation with protons did not affect the values of Young’s modulus.

**Figure 7:**
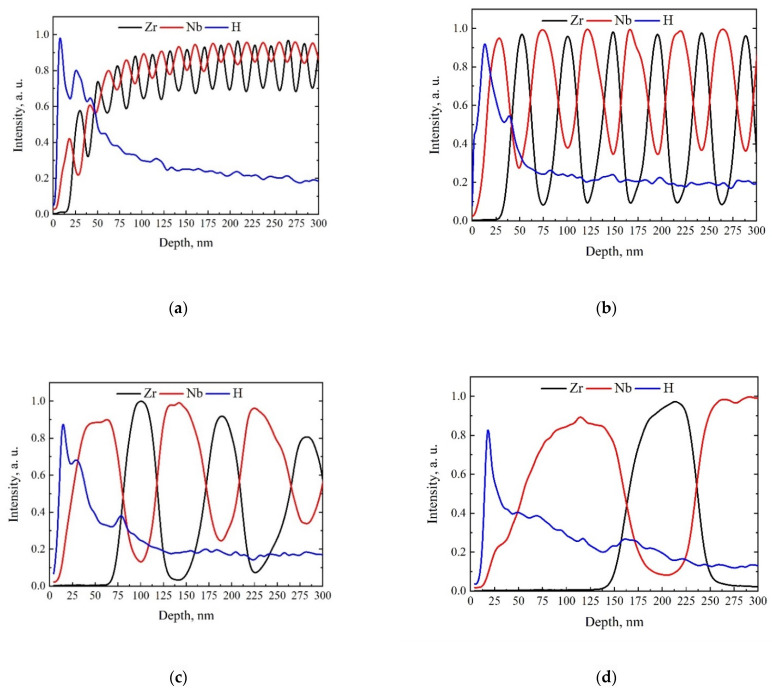
Depth distribution of elements for Zr/Nb NMCs after proton irradiation for: Zr/Nb 10 nm (**a**), Zr/Nb 25 nm (**b**), Zr/Nb 50 nm (**c**), and Zr/Nb 100 nm (**d**).

Figure 7 shows the distribution of elements after H^+^ ion irradiation for Zr/Nb NMCs in an irradiation region up to 300 nm.

It can be seen from Figure 7 that all irradiated Zr/Nb NMCs with individual layer thicknesses from 25 to 100 nm were characterized by the local H maximum at the Nb/Zr interfaces, while at the Zr/Nb interfaces, abundant H accumulation was not observed, and its level corresponded to the Bragg peak distribution. This effect was previously observed during proton implantation at ~800 nm in Zr/Nb 100/100 nm but in a more pronounced form [46]. The 10 nm system had a different H distribution. In the first three layers, H accumulation occurred predominantly within the layers rather than at the interfaces. This is possibly due to radiation destruction of the interface structure, which leads to changes in the sink strength or diffusion properties of the system. In subsequent layers, the irregular H accumulation at the interfaces generally persisted. A possible reason for this consists of proton diffusion behavior in the bulk of Zr and Nb multilayers as well as near the interface’s region.

**Figure 8:**
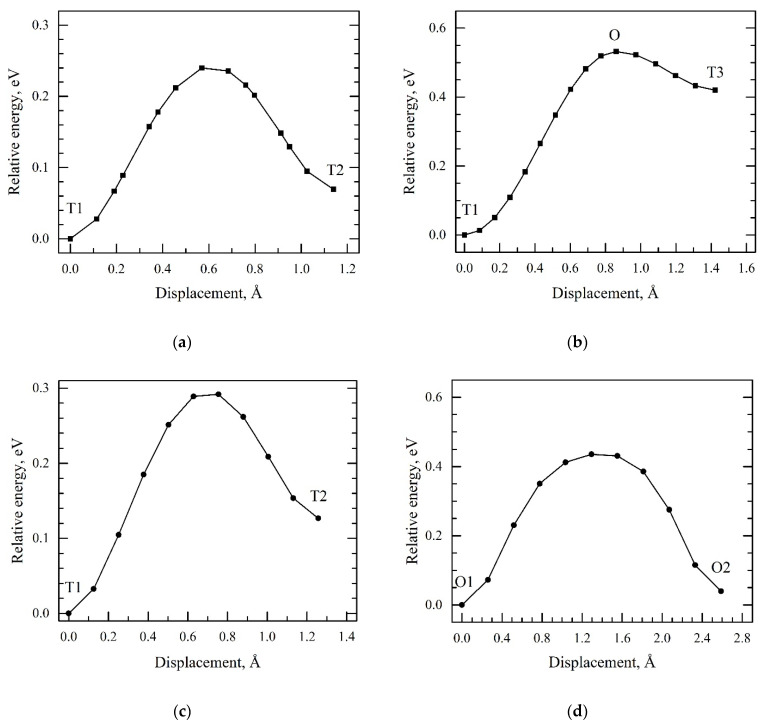

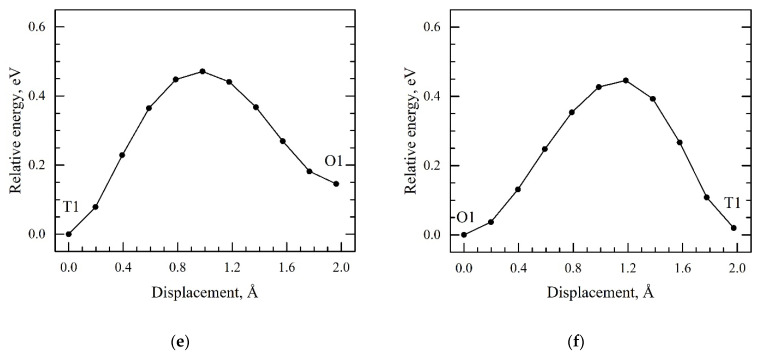
The minimum energy paths for hydrogen atoms along with the directions of (**a**) T1-T2 and (**b**) T1-O-T3 diffusion jumps in the BCC niobium lattice and (**c**) T1-T2, (**d**) O1-O2, (**e**) T1-O1, and (**f**) O1-T1 in the HCP zirconium lattice.

To explain the H diffusion behavior in the Zr/Nb NMCs, the minimum energy path for different hydrogen diffusion jumps in the Zr and Nb layers and the Nb/Zr interface was calculated from the first principles. Figure 8 shows the diffusion barrier energy path calculated for the hydrogen atom in the BCC niobium and HCP zirconium lattice. The results demonstrate a relatively good qualitative agreement with the results of other calculations for Nb [57,58] and Zr [47,48]. In niobium, the T1-T2 diffusion barrier was significantly lower than the T1-O1-T3 diffusion barrier. As a result, a hydrogen atom migrated predominantly through tetrahedral interstitial sites in the Nb bulk. In zirconium, the minimum barrier corresponded to the T1-T2 jumps, and the maximum of the calculated ones was T1-O1. Figure 1b shows that the T1-T2 jump did not allow the hydrogen atom to migrate throughout the Zr bulk. The main diffusion pathways were the T1-O1 and O1-T1 jumps and O1-O2 jumps along the hexagonal axis of the Zr HCP lattice. All these diffusion barriers were significantly higher than the T1-T2 diffusion barrier in niobium.

**Figure 9:**
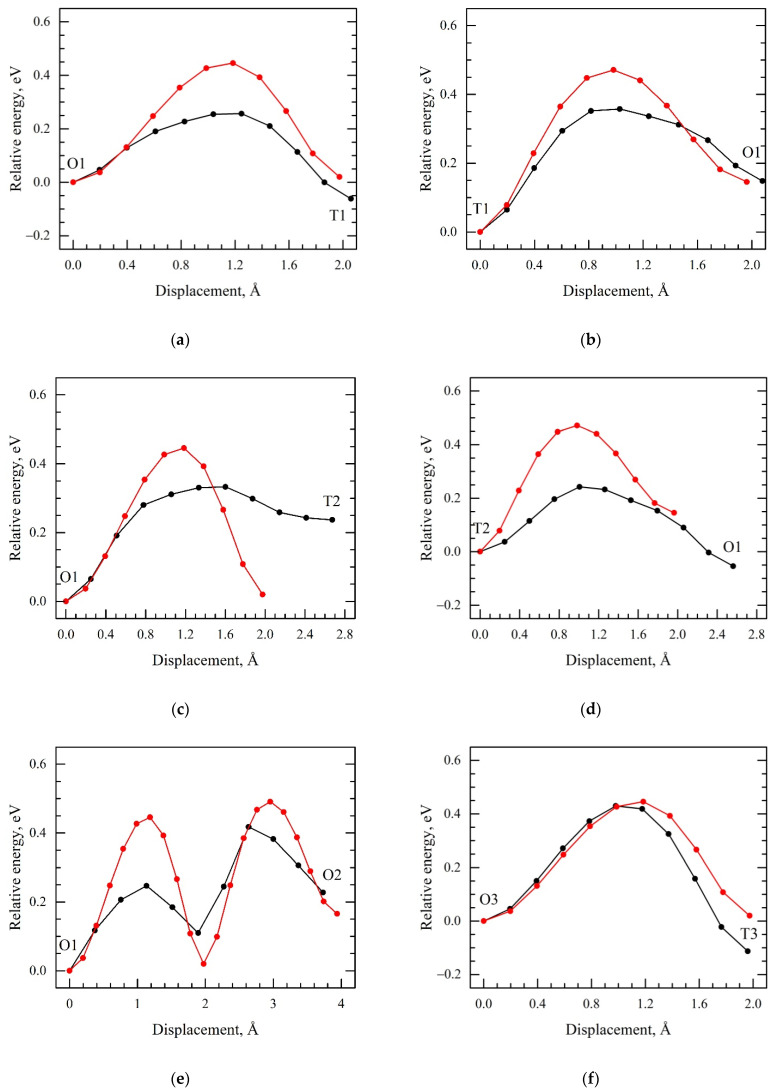
The minimum energy paths for a hydrogen atom along the directions of (**a**) O1-T1, (**b**) T1-O1, (**c**) O1-T2, (**d**) T2-O1, (**e**) O1-O2, and (**f**) T3-O3 in the Zr atomic layers of Zr_36_Nb_36_ slab nearest to the interface (black line) and the same diffusion energy paths for hydrogen atoms in the HCP zirconium lattice (red line).

The next step of the current study was to calculate the diffusion barriers in the zirconium atomic layer near the interface of the Zr/Nb multilayer structure and to compare the results with the corresponding diffusion barriers in the zirconium lattice. The minimum energy paths calculated for hydrogen atoms near the interface of the Zr_36_Nb_36_ slab are presented in Figure 9. Analysis of these results showed that the length of diffusion jumps for most of the cases considered in the zirconium layer of the Zr_36_Nb_36_ slab was less than in pure zirconium. This is caused by the strong distortion of the zirconium lattice (a significant shift in zirconium atoms toward the interface) due to the relaxation near the interface [46]. A decrease in the height of the diffusion barriers reached 44% near the Zr/Nb interface. As a result, hydrogen diffusion near the interface was more efficient, compared with the Zr bulk and comparable to that in the Nb bulk.

**Figure 10:**
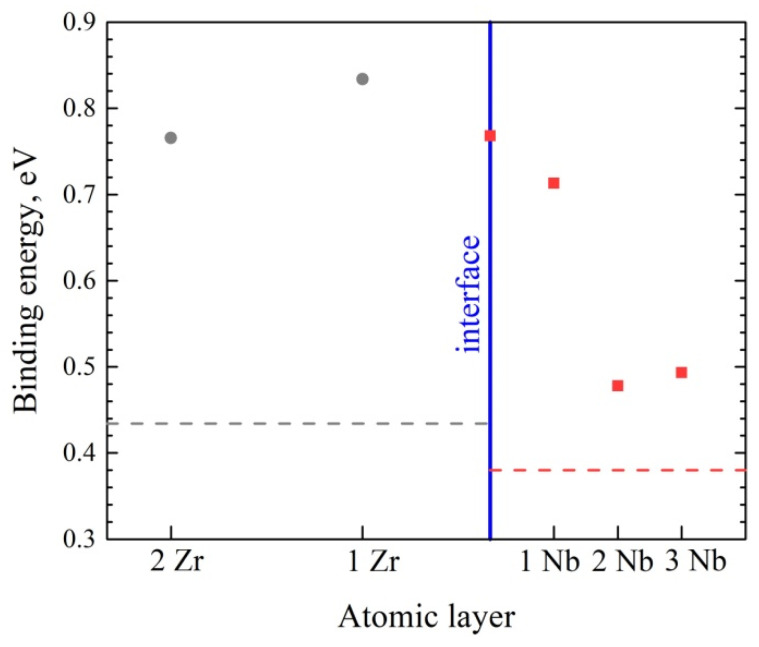
The dependence of the average value of hydrogen binding energy on the atomic layer number. The red and gray dotted lines indicate the average value of hydrogen binding energy in the Nb and Zr bulk, respectively.

It was shown previously [46] that the average value of hydrogen binding energy per the metal atomic layer decreased with an increase in the distance between the atomic layer and interface (Figure 10). Moreover, in the Zr layer, this decrease was slower than in the Nb layers. The irregular accumulation of hydrogen in the Zr/Nb NMCs in Figure 7 is explained by the two factors. The first of these is the fast hydrogen diffusion in the Nb layers, which leads to the migration of hydrogen atoms from the bulk to the interface according to the classical Fick’s law, where these atoms are captured and distributed along the interface. The second factor is the low hydrogen diffusion in the Zr layers, which leads to the hydrogen distribution in the Zr bulk according to the Bragg peak.

**Figure 11:**
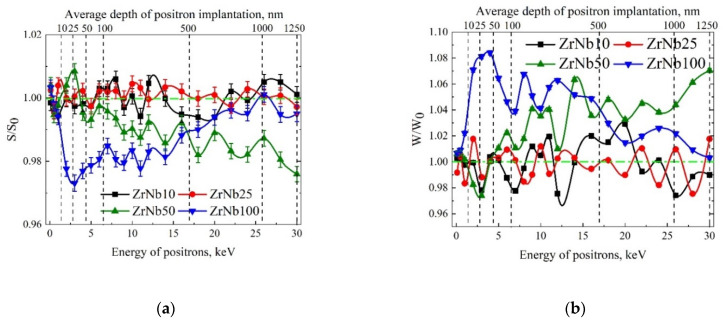
Dependence of the relative S and W parameter on the positron energy S/S_0_ = f(E) (**a**) and W/W_0_ = f(E) (**b**) for Zr/Nb NMCs after irradiation.

Figure 11 shows the dependence of the relative parameters S/S_0_ and W/W_0_ on the positron energy for Zr/Nb NMCs after irradiation, where S_0_ and W_0_ are parameters before irradiation, and S and W are parameters after irradiation.

As is seen in Figure 11, the character of the change in the annihilation parameters does not differ significantly from our previous experiments [38,46]. When the thickness of an individual layer was much less than the average diffusion length, which, in nanomaterials, is usually comparable to the size of nanocrystals (~32 ± 15 nm in this case), positrons were predominantly annihilated in zirconium sublattices. This is due to the greater affinity of zirconium for positrons, as well as the presence of regions of reduced electron density at the interface from the side of zirconium [46]. Therefore, the values of S/S_0_ and W/W_0_ parameters close to unity were similar to Zr for NMCs, even at low positron energies. As can be observed, the protons irradiation did not lead to an increase in S and a decrease in W parameters in Zr/Nb NMCs, with thicknesses of individual layers of 10 nm and 25 nm. Only minor changes in the shape of the DBS parameters occurred, which can be explained based on the experimental error. This indicates the lack of structural changes in these layers. However, such changes were observed for Zr/Nb with thicknesses of 50 nm and 100 nm. According to DBS data, these systems were characterized by a lower relative S/S_0_ value and higher W/W_0_, which usually indicates a free volume reduction as a result of defect annealing. In this case, in Zr/Nb NMCs with 50 nm layer thicknesses, the most significant changes occurred in the deeper layers (~100 ÷ 1000 nm), and in Zr/Nb, NMCs with 100 nm layer thicknesses, they occurred in ~10 ÷ 700 nm deep range. The physical reason for this effect remains unclear and requires further investigation. Changes in the relative DBS parameters were caused by both the microstructural features of Zr/Nb NMCs and the particularities of positron annihilation in these systems.

## 4. Conclusions

As a result of studying the effect of proton irradiation on the microstructure, structural and phase state, mechanical properties, and defect state, the following findings were revealed:

(1) Irradiation at the studied doses did not affect the crystal structure and grain orientation inside the Zr and Nb layers. A feature of the samples with a thickness of individual Zr/Nb layers of 10 nm was the presence of nanovoids consisting of several vacant atomic positions. After irradiation, slight changes in the diffraction pattern were noted for Zr/Nb NMCs with a separate layer thickness of 50 and 100 nm. The shift in the diffraction peak in irradiated metal multilayers was caused by the crystal lattice distortions and stresses arising in the layers.
(2) An increase in the thickness of alternating Zr/Nb multilayers from 25 to 100 nm led to a significant decrease in the nanohardness values of the samples, compared with NMC samples with a thickness of individual Zr/Nb layers of 10 nm. Proton irradiation at the studied parameters led to a decrease in the values of nanohardness of the Zr/Nb NMCs. Meanwhile, irradiation with protons did not affect the values of Young’s modulus.
(3) The layer-by-layer analysis by DBS using a variable-energy positron beam did not reveal the additional defects in NMC Zr/Nb after proton irradiation. The S parameter remained unchanged or decreased.
(4) The irregular H accumulation at the interfaces in NMC Zr/Nb after proton irradiation was observed; H accumulated at the Nb/Zr interface much more than at the Zr/Nb interface possibly due to significant differences in H diffusion barriers in the bulk of Zr and Nb multilayers, as well as near the interface’s region.
(5) The average value of hydrogen binding energy decreased with an increase in the distance between the atomic layer and interface slower in the Zr layer than in the Nb layer. Hydrogen diffusion along the interface was more efficient than in the Zr bulk and comparable to diffusion in the Nb bulk.

## Data Availability

Data are contained within the article.
